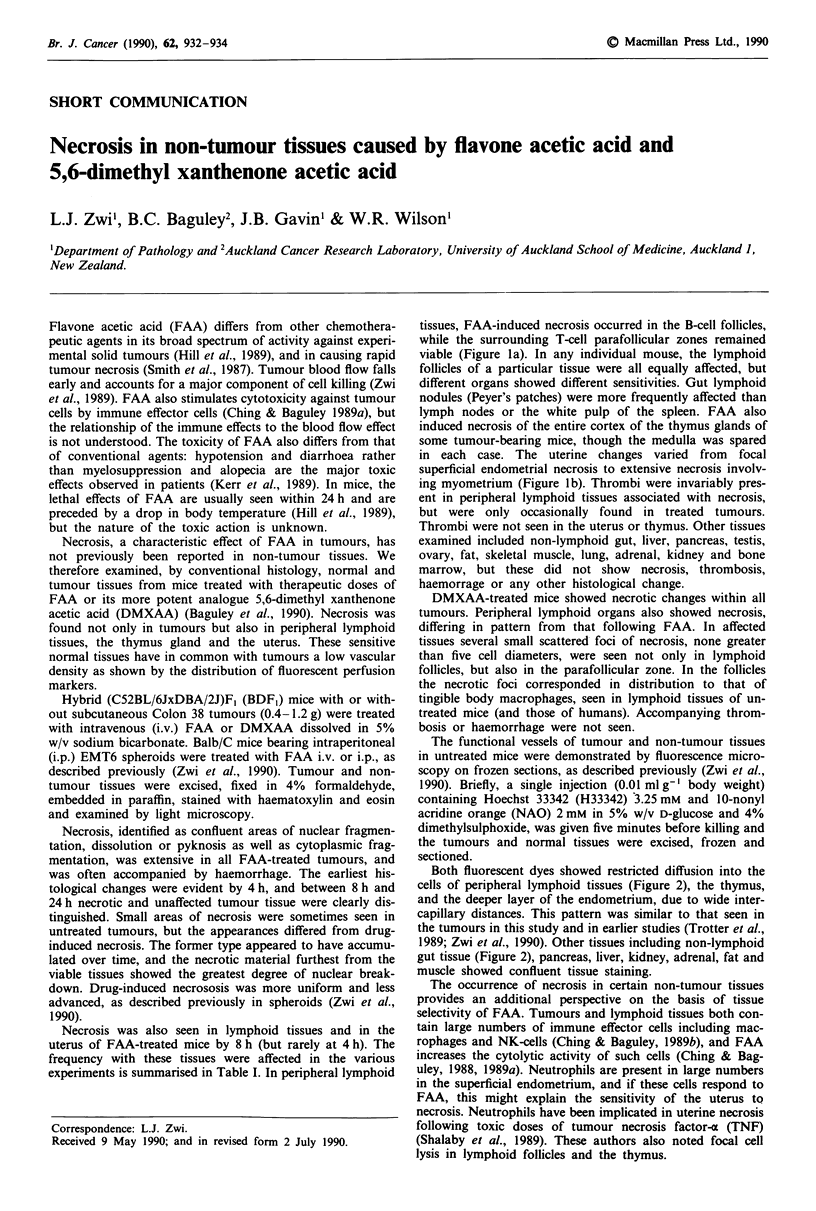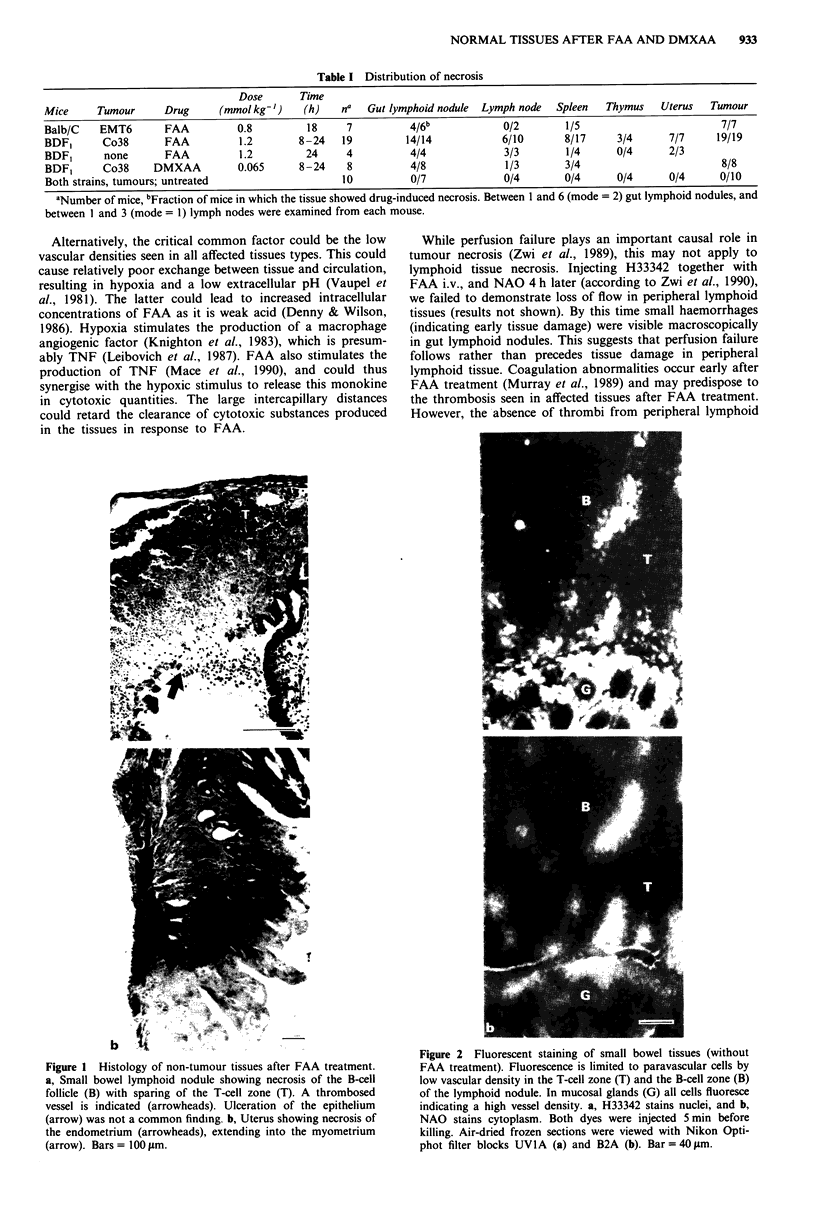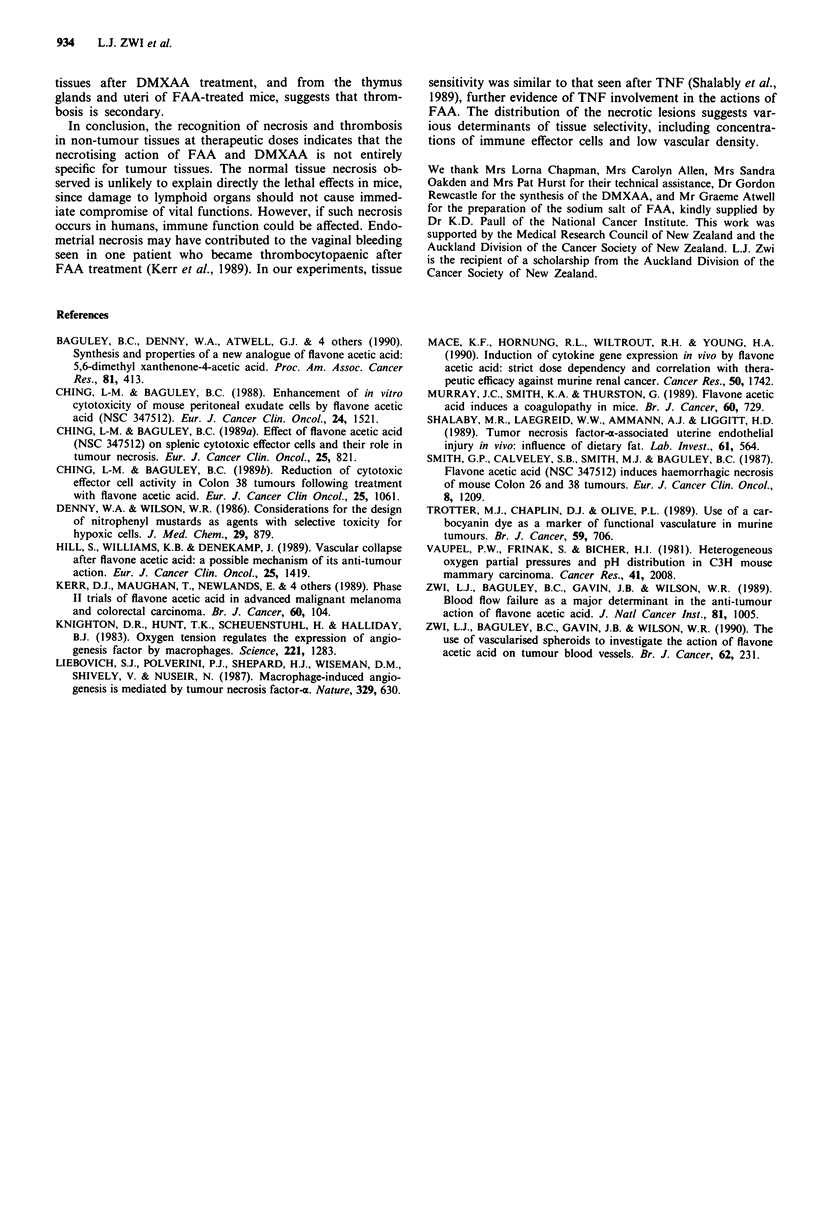# Necrosis in non-tumour tissues caused by flavone acetic acid and 5,6-dimethyl xanthenone acetic acid.

**DOI:** 10.1038/bjc.1990.412

**Published:** 1990-12

**Authors:** L. J. Zwi, B. C. Baguley, J. B. Gavin, W. R. Wilson

**Affiliations:** Department of Pathology, University of Auckland School of Medicine, New Zealand.

## Abstract

**Images:**


Br. J. Cancer (1990), 62, 932-934 © Macmillan Press Ltd., 1990

SHORT COMMUNICATION

### Necrosis in non-tumour tissues caused by flavone acetic acid and 5,6-dimethyl xanthenone acetic acid

L.J. Zwi[1], B.C. Baguley[2], J.B. Gavin[1] & W.R. Wilson[1]

[1]Department of Pathology and [2]Auckland Cancer Research Laboratory, University of Auckland School of Medicine, Auckland 1, New Zealand.

Flavone acetic acid (FAA) differs from other chemotherapeutic agents in its broad spectrum of activity against experimental solid tumours (Hill *et al.*, 1989), and in causing rapid tumour necrosis (Smith *et al.*, 1987). Tumour blood flow falls early and accounts for a major component of cell killing (Zwi *et al.*, 1989). FAA also stimulates cytotoxicity against tumour cells by immune effector cells (Ching & Baguley 1989*a*), but the relationship of the immune effects to the blood flow effect is not understood. The toxicity of FAA also differs from that of conventional agents: hypotension and diarrhoea rather than myelosuppression and alopecia are the major toxic effects observed in patients (Kerr *et al.*, 1989). In mice, the lethal effects of FAA are usually seen within 24 h and are preceded by a drop in body temperature (Hill *et al.*, 1989), but the nature of the toxic action is unknown.

Necrosis, a characteristic effect of FAA in tumours, has not previously been reported in non-tumour tissues. We therefore examined, by conventional histology, normal and tumour tissues from mice treated with therapeutic doses of FAA or its more potent analogue 5,6-dimethyl xanthenone acetic acid (DMXAA) (Baguley *et al.*, 1990). Necrosis was found not only in tumours but also in peripheral lymphoid tissues, the thymus gland and the uterus. These sensitive normal tissues have in common with tumours a low vascular density as shown by the distribution of fluorescent perfusion markers.

Hybrid (C52BL/6JxDBA/2J)$F_1$ ($BDF_1$) mice with or without subcutaneous Colon 38 tumours (0.4–1.2 g) were treated with intravenous (i.v.) FAA or DMXAA dissolved in 5% w/v sodium bicarbonate. Balb/C mice bearing intraperitoneal (i.p.) EMT6 spheroids were treated with FAA i.v. or i.p., as described previously (Zwi *et al.*, 1990). Tumour and non-tumour tissues were excised, fixed in 4% formaldehyde, embedded in paraffin, stained with haematoxylin and eosin and examined by light microscopy.

Necrosis, identified as confluent areas of nuclear fragmentation, dissolution or pyknosis as well as cytoplasmic fragmentation, was extensive in all FAA-treated tumours, and was often accompanied by haemorrhage. The earliest histological changes were evident by 4 h, and between 8 h and 24 h necrotic and unaffected tumour tissue were clearly distinguished. Small areas of necrosis were sometimes seen in untreated tumours, but the appearances differed from drug-induced necrosis. The former type appeared to have accumulated over time, and the necrotic material furthest from the viable tissues showed the greatest degree of nuclear breakdown. Drug-induced necrososis was more uniform and less advanced, as described previously in spheroids (Zwi *et al.*, 1990).

Necrosis was also seen in lymphoid tissues and in the uterus of FAA-treated mice by 8 h (but rarely at 4 h). The frequency with these tissues were affected in the various experiments is summarised in Table I. In peripheral lymphoid tissues, FAA-induced necrosis occurred in the B-cell follicles, while the surrounding T-cell parafollicular zones remained viable (Figure 1a). In any individual mouse, the lymphoid follicles of a particular tissue were all equally affected, but different organs showed different sensitivities. Gut lymphoid nodules (Peyer's patches) were more frequently affected than lymph nodes or the white pulp of the spleen. FAA also induced necrosis of the entire cortex of the thymus glands of some tumour-bearing mice, though the medulla was spared in each case. The uterine changes varied from focal superficial endometrial necrosis to extensive necrosis involving myometrium (Figure 1b). Thrombi were invariably present in peripheral lymphoid tissues associated with necrosis, but were only occasionally found in treated tumours. Thrombi were not seen in the uterus or thymus. Other tissues examined included non-lymphoid gut, liver, pancreas, testis, ovary, fat, skeletal muscle, lung, adrenal, kidney and bone marrow, but these did not show necrosis, thrombosis, haemorrage or any other histological change.

DMXAA-treated mice showed necrotic changes within all tumours. Peripheral lymphoid organs also showed necrosis, differing in pattern from that following FAA. In affected tissues several small scattered foci of necrosis, none greater than five cell diameters, were seen not only in lymphoid follicles, but also in the parafollicular zone. In the follicles the necrotic foci corresponded in distribution to that of tingible body macrophages, seen in lymphoid tissues of untreated mice (and those of humans). Accompanying thrombosis or haemorrhage were not seen.

The functional vessels of tumour and non-tumour tissues in untreated mice were demonstrated by fluorescence microscopy on frozen sections, as described previously (Zwi *et al.*, 1990). Briefly, a single injection (0.01 ml $g^{-1}$ body weight) containing Hoechst 33342 (H33342) 3.25 mM and 10-nonyl acridine orange (NAO) 2 mM in 5% w/v D-glucose and 4% dimethylsulphoxide, was given five minutes before killing and the tumours and normal tissues were excised, frozen and sectioned.

Both fluorescent dyes showed restricted diffusion into the cells of peripheral lymphoid tissues (Figure 2), the thymus, and the deeper layer of the endometrium, due to wide intercapillary distances. This pattern was similar to that seen in the tumours in this study and in earlier studies (Trotter *et al.*, 1989; Zwi *et al.*, 1990). Other tissues including non-lymphoid gut tissue (Figure 2), pancreas, liver, kidney, adrenal, fat and muscle showed confluent tissue staining.

The occurrence of necrosis in certain non-tumour tissues provides an additional perspective on the basis of tissue selectivity of FAA. Tumours and lymphoid tissues both contain large numbers of immune effector cells including macrophages and NK-cells (Ching & Baguley, 1989*b*), and FAA increases the cytolytic activity of such cells (Ching & Baguley, 1988, 1989*a*). Neutrophils are present in large numbers in the superficial endometrium, and if these cells respond to FAA, this might explain the sensitivity of the uterus to necrosis. Neutrophils have been implicated in uterine necrosis following toxic doses of tumour necrosis factor-α (TNF) (Shalaby *et al.*, 1989). These authors also noted focal cell lysis in lymphoid follicles and the thymus.

Correspondence: L.J. Zwi.
Received 9 May 1990; and in revised form 2 July 1990.

**Table I** Distribution of necrosis

| *Mice* | *Tumour* | *Drug* | *Dose (mmol kg$^{-1}$)* | *Time (h)* | $n^a$ | *Gut lymphoid nodule* | *Lymph node* | *Spleen* | *Thymus* | *Uterus* | *Tumour* |
|---|---|---|---|---|---|---|---|---|---|---|---|
| Balb/C | EMT6 | FAA | 0.8 | 18 | 7 | 4/6[b] | 0/2 | 1/5 | | | 7/7 |
| $BDF_1$ | Co38 | FAA | 1.2 | 8–24 | 19 | 14/14 | 6/10 | 8/17 | 3/4 | 7/7 | 19/19 |
| $BDF_1$ | none | FAA | 1.2 | 24 | 4 | 4/4 | 3/3 | 1/4 | 0/4 | 2/3 | |
| $BDF_1$ | Co38 | DMXAA | 0.065 | 8–24 | 8 | 4/8 | 1/3 | 3/4 | | | 8/8 |
| Both strains, tumours; untreated | | | | | 10 | 0/7 | 0/4 | 0/4 | 0/4 | 0/4 | 0/10 |

[a]Number of mice, [b]Fraction of mice in which the tissue showed drug-induced necrosis. Between 1 and 6 (mode = 2) gut lymphoid nodules, and between 1 and 3 (mode = 1) lymph nodes were examined from each mouse.

Alternatively, the critical common factor could be the low vascular densities seen in all affected tissues types. This could cause relatively poor exchange between tissue and circulation, resulting in hypoxia and a low extracellular pH (Vaupel *et al.*, 1981). The latter could lead to increased intracellular concentrations of FAA as it is weak acid (Denny & Wilson, 1986). Hypoxia stimulates the production of a macrophage angiogenic factor (Knighton *et al.*, 1983), which is presumably TNF (Leibovich *et al.*, 1987). FAA also stimulates the production of TNF (Mace *et al.*, 1990), and could thus synergise with the hypoxic stimulus to release this monokine in cytotoxic quantities. The large intercapillary distances could retard the clearance of cytotoxic substances produced in the tissues in response to FAA.

While perfusion failure plays an important causal role in tumour necrosis (Zwi *et al.*, 1989), this may not apply to lymphoid tissue necrosis. Injecting H33342 together with FAA i.v., and NAO 4 h later (according to Zwi *et al.*, 1990), we failed to demonstrate loss of flow in peripheral lymphoid tissues (results not shown). By this time small haemorrhages (indicating early tissue damage) were visible macroscopically in gut lymphoid nodules. This suggests that perfusion failure follows rather than precedes tissue damage in peripheral lymphoid tissue. Coagulation abnormalities occur early after FAA treatment (Murray *et al.*, 1989) and may predispose to the thrombosis seen in affected tissues after FAA treatment. However, the absence of thrombi from peripheral lymphoid

**Figure 1** Histology of non-tumour tissues after FAA treatment. **a**, Small bowel lymphoid nodule showing necrosis of the B-cell follicle (B) with sparing of the T-cell zone (T). A thrombosed vessel is indicated (arrowheads). Ulceration of the epithelium (arrow) was not a common finding. **b**, Uterus showing necrosis of the endometrium (arrowheads), extending into the myometrium (arrow). Bars = 100 μm.

**Figure 2** Fluorescent staining of small bowel tissues (without FAA treatment). Fluorescence is limited to paravascular cells by low vascular density in the T-cell zone (T) and the B-cell zone (B) of the lymphoid nodule. In mucosal glands (G) all cells fluoresce indicating a high vessel density. **a**, H33342 stains nuclei, and **b**, NAO stains cytoplasm. Both dyes were injected 5 min before killing. Air-dried frozen sections were viewed with Nikon Optiphot filter blocks UV1A (**a**) and B2A (**b**). Bar = 40 μm.

tissues after DMXAA treatment, and from the thymus glands and uteri of FAA-treated mice, suggests that thrombosis is secondary.

In conclusion, the recognition of necrosis and thrombosis in non-tumour tissues at therapeutic doses indicates that the necrotising action of FAA and DMXAA is not entirely specific for tumour tissues. The normal tissue necrosis observed is unlikely to explain directly the lethal effects in mice, since damage to lymphoid organs should not cause immediate compromise of vital functions. However, if such necrosis occurs in humans, immune function could be affected. Endometrial necrosis may have contributed to the vaginal bleeding seen in one patient who became thrombocytopaenic after FAA treatment (Kerr *et al.*, 1989). In our experiments, tissue sensitivity was similar to that seen after TNF (Shalaby *et al.*, 1989), further evidence of TNF involvement in the actions of FAA. The distribution of the necrotic lesions suggests various determinants of tissue selectivity, including concentrations of immune effector cells and low vascular density.

We thank Mrs Lorna Chapman, Mrs Carolyn Allen, Mrs Sandra Oakden and Mrs Pat Hurst for their technical assistance, Dr Gordon Rewcastle for the synthesis of the DMXAA, and Mr Graeme Atwell for the preparation of the sodium salt of FAA, kindly supplied by Dr K.D. Paull of the National Cancer Institute. This work was supported by the Medical Research Council of New Zealand and the Auckland Division of the Cancer Society of New Zealand. L.J. Zwi is the recipient of a scholarship from the Auckland Division of the Cancer Society of New Zealand.

### References

BAGULEY, B.C., DENNY, W.A., ATWELL, G.J. & 4 others (1990). Synthesis and properties of a new analogue of flavone acetic acid: 5,6-dimethyl xanthenone-4-acetic acid. *Proc. Am. Assoc. Cancer Res.*, **81**, 413.

CHING, L-M. & BAGULEY, B.C. (1988). Enhancement of *in vitro* cytotoxicity of mouse peritoneal exudate cells by flavone acetic acid (NSC 347512). *Eur. J. Cancer Clin. Oncol.*, **24**, 1521.

CHING, L-M. & BAGULEY, B.C. (1989*a*). Effect of flavone acetic acid (NSC 347512) on splenic cytotoxic effector cells and their role in tumour necrosis. *Eur. J. Cancer Clin. Oncol.*, **25**, 821.

CHING, L-M. & BAGULEY, B.C. (1989*b*). Reduction of cytotoxic effector cell activity in Colon 38 tumours following treatment with flavone acetic acid. *Eur. J. Cancer Clin Oncol.*, **25**, 1061.

DENNY, W.A. & WILSON, W.R. (1986). Considerations for the design of nitrophenyl mustards as agents with selective toxicity for hypoxic cells. *J. Med. Chem.*, **29**, 879.

HILL, S., WILLIAMS, K.B. & DENEKAMP, J. (1989). Vascular collapse after flavone acetic acid: a possible mechanism of its anti-tumour action. *Eur. J. Cancer Clin. Oncol.*, **25**, 1419.

KERR, D.J., MAUGHAN, T., NEWLANDS, E. & 4 others (1989). Phase II trials of flavone acetic acid in advanced malignant melanoma and colorectal carcinoma. *Br. J. Cancer*, **60**, 104.

KNIGHTON, D.R., HUNT, T.K., SCHEUENSTUHL, H. & HALLIDAY, B.J. (1983). Oxygen tension regulates the expression of angiogenesis factor by macrophages. *Science*, **221**, 1283.

LIEBOVICH, S.J., POLVERINI, P.J., SHEPARD, H.J., WISEMAN, D.M., SHIVELY, V. & NUSEIR, N. (1987). Macrophage-induced angiogenesis is mediated by tumour necrosis factor-α. *Nature*, **329**, 630.

MACE, K.F., HORNUNG, R.L., WILTROUT, R.H. & YOUNG, H.A. (1990). Induction of cytokine gene expression *in vivo* by flavone acetic acid: strict dose dependency and correlation with therapeutic efficacy against murine renal cancer. *Cancer Res.*, **50**, 1742.

MURRAY, J.C., SMITH, K.A. & THURSTON, G. (1989). Flavone acetic acid induces a coagulopathy in mice. *Br. J. Cancer*, **60**, 729.

SHALABY, M.R., LAEGREID, W.W., AMMANN, A.J. & LIGGITT, H.D. (1989). Tumor necrosis factor-α-associated uterine endothelial injury *in vivo*: influence of dietary fat. *Lab. Invest.*, **61**, 564.

SMITH, G.P., CALVELEY, S.B., SMITH, M.J. & BAGULEY, B.C. (1987). Flavone acetic acid (NSC 347512) induces haemorrhagic necrosis of mouse Colon 26 and 38 tumours. *Eur. J. Cancer Clin. Oncol.*, **8**, 1209.

TROTTER, M.J., CHAPLIN, D.J. & OLIVE, P.L. (1989). Use of a carbocyanin dye as a marker of functional vasculature in murine tumours. *Br. J. Cancer*, **59**, 706.

VAUPEL, P.W., FRINAK, S. & BICHER, H.I. (1981). Heterogeneous oxygen partial pressures and pH distribution in C3H mouse mammary carcinoma. *Cancer Res.*, **41**, 2008.

ZWI, L.J., BAGULEY, B.C., GAVIN, J.B. & WILSON, W.R. (1989). Blood flow failure as a major determinant in the anti-tumour action of flavone acetic acid. *J. Natl Cancer Inst.*, **81**, 1005.

ZWI, L.J., BAGULEY, B.C., GAVIN, J.B. & WILSON, W.R. (1990). The use of vascularised spheroids to investigate the action of flavone acetic acid on tumour blood vessels. *Br. J. Cancer*, **62**, 231.